# Hypothyroidism and Hyperthyroidism in an Adolescent With Complex Congenital Heart Disease Exposed to Iodinated Contrast Media: Case Report

**DOI:** 10.1155/crie/6859739

**Published:** 2025-07-15

**Authors:** Elizabeth S. Sandberg, Jacqueline S. Fisher

**Affiliations:** ^1^Department of Pediatrics, Division of Endocrinology, C.S. Mott Children's Hospital, University of Michigan, Ann Arbor, Michigan, USA; ^2^Children's Minnesota Division of Pediatric Endocrinology, Saint Paul, Minnesota, USA

**Keywords:** case report, complex congenital heart disease, hyperthyroidism, hypothyroidism, Jod-Basedow

## Abstract

**Introduction:** Jod-Basedow syndrome and iodine-induced hypothyroidism are rare but clinically significant complications of iodine exposure. We report a unique case of a 14-year-old boy with congenital heart disease, who developed sequential iodine-induced hypothyroidism due to failure to escape from the Wolff–Chaikoff effect, followed by hyperthyroidism (Jod-Basedow syndrome) after additional exposure to iodinated contrast.

**Case Presentation:** The patient is a 14-year-old male with a history of complex congenital cardiac defects. He underwent a Fontan procedure to manage his single-ventricle physiology, resulting in plastic bronchitis requiring lymphatic intervention and cardiac catheterization. At age 15, he developed hypothyroidism requiring levothyroxine, but 6 months later presented with symptoms and labs confirming hyperthyroidism following CT scan with IV iodinated contrast. Levothyroxine was discontinued, and methimazole was initiated to manage his hyperthyroidism.

**Conclusion:** Iodine-induced hypothyroidism and Jod-Basedow syndrome should be considered potential complications for patients who undergo iodine exposure. This case highlights the importance of vigilant thyroid monitoring in congenital heart disease patients who are undergoing frequent radiation and iodine contrast exposure. In this patient, both his hypothyroidism and hyperthyroidism were successfully managed, but his overall condition deteriorated, ultimately requiring a heart transplant. This case underscores the importance of close monitoring of thyroid hormone levels in complex cardiac patients who undergo repeated exposure to iodinated contrast during procedures and imaging studies.

## 1. Introduction

Both iodine-induced hypothyroidism and hyperthyroidism are recognized consequences of iodine excess. Hypothyroidism may result from failure to escape the Wolff–Chaikoff effect, while hyperthyroidism may arise from the Jod-Basedow phenomenon. We present a unique case of a 15-year-old boy with a history of complex congenital heart disease, who developed both conditions after sequential exposures to iodinated contrast media, first hypothyroidism, then hyperthyroidism, illustrating the spectrum of iodine-induced thyroid dysfunction in a single patient.

## 2. Patient Information

A 14-year-old male with a history of congenital heart defects, including double outlet right ventricle (DORV), hypoplastic right ventricle, interrupted aortic arch, and bilateral patent ductus arteriosus (PDA) presented to pediatric endocrinology initially with concern for hypothyroidism. His past medical history was significant for undergoing a Fontan procedure when he was 2 years old. This was performed to manage his single-ventricle physiology, and he subsequently developed plastic bronchitis, a known post-Fontan complication causing abnormal flow of lymph. He underwent lymphatic intervention to manage the plastic bronchitis and associated cardiac catheterization using iodinated-contrast. Due to complications of his plastic bronchitis, this lymphatic intervention and associated cardiac catheterization were repeated 1 month later. All dynamic imaging was done using MRI and gadolinium-based contrast.

### 2.1. Initial Presentation and Hypothyroidism Diagnosis

One year after lymphatic intervention, the now 14-year-old male presented to pediatric endocrinology with worsening fatigue, increased daytime napping, migraines, slowed speech, and facial swelling. On examination, he was noted to have slowed speech and a puffy face, raising concern for hypothyroidism. Laboratory evaluation revealed TSH >150 IU/mL (normal range 0.30–5.50 mIU/L), Free T4 0.52 ng/dL (normal range 0.76–1.70 ng/dL), and negative thyroid peroxidase (TPO) and thyroglobulin antibodies.

**Figure 1:**
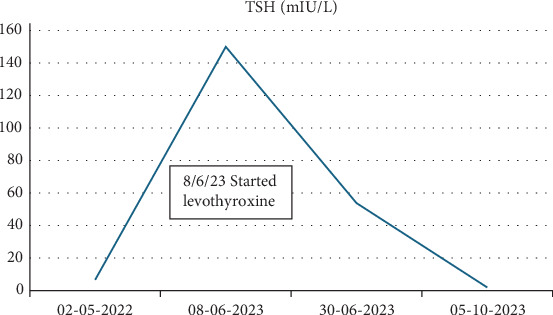
TSH during hypothyroidism diagnosis and treatment.

**Figure 2:**
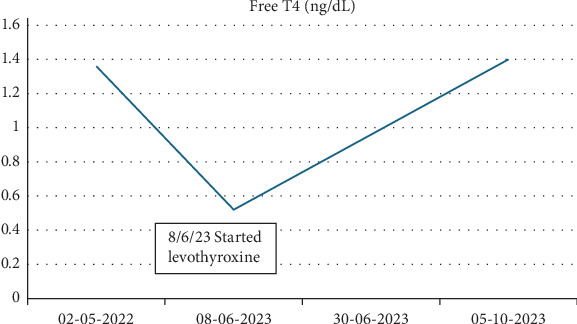
Free T4 during hypothyroidism diagnosis and treatment.

Given his negative autoantibodies and no known exposure to other thyroid-disrupting agents, such as lithium or amiodarone, his hypothyroidism was attributed to failure to escape from the Wolff–Chaikoff effect following iodine exposure during his cardiac catheterization the previous year. The patient was started on levothyroxine 50 µg daily, with close monitoring of thyroid function after achieving a euthyroid state (Figures 1 and 2).

### 2.2. Development of Iodine-Induced Hyperthyroidism (Jod-Basedow Syndrome)

Six months after initiating thyroid hormone replacement, he was admitted with worsening plastic bronchitis. He underwent a CT chest with iodinated contrast to rule out pulmonary embolism. Two days later, he developed tachycardia and bilateral hand tremor. Thyroid studies were obtained, revealing a suppressed TSH of 0.01 mIU/L and elevated free T4 of 5.25 ng/dL.

Given symptoms and laboratory confirmation of hyperthyroidism, levothyroxine was discontinued. The family verified that the appropriate levothyroxine dose was being administered, and presentation was not consistent with medication overdose. Graves' disease was ruled out, as thyroid-stimulating immunoglobulin (TSI) and thyrotropin receptor antibodies were normal. Thyroid ultrasound showed a mildly enlarged heterogenous and hypervascular thyroid gland and no nodules. Thyroid uptake scan to rule out a hyperfunctioning adenoma was not possible due to exposure to iodinated contrast from recent CT.

**Figure 3:**
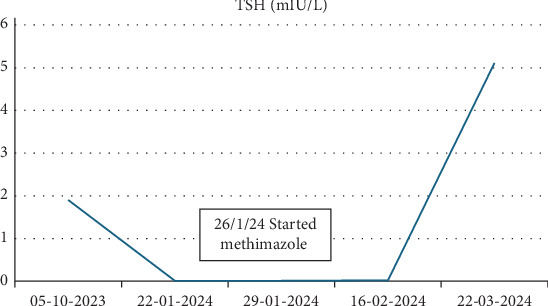
TSH during hyperthyroidism diagnosis and treatment.

**Figure 4:**
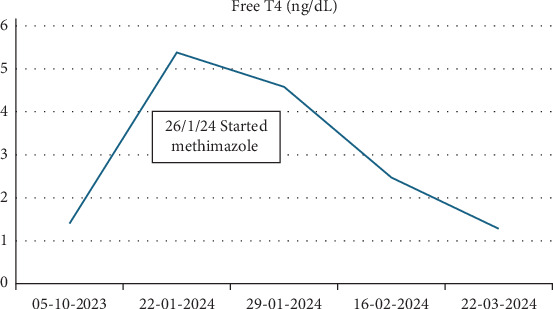
Free T4 during hyperthyroidism diagnosis and treatment.

He had persistent hyperthyroidism despite discontinuation of levothyroxine. Methimazole was initiated at 5 mg BID and was escalated to a total daily dose of 25 mg, with close monitoring of thyroid function and cardiac status given his Fontan physiology. One month after starting methimazole, the dose was reduced in response to normalized T3/T4 levels, and methimazole was ultimately discontinued after 6 weeks of treatment (Figures 3 and 4). He has remained euthyroid since that time.

His change from a hypothyroid to hyperthyroid state was consistent with iatrogenic hyperthyroidism, concerning for Jod-Basedow syndrome. Given his recent iodine exposure during CT chest with contrast in the setting of known thyroid dysfunction, iodine-induced hyperthyroidism was suspected. This patient's progression from iodine-induced hypothyroidism to hyperthyroidism illustrates the dynamic nature of thyroid iodine handling.

### 2.3. Progression to Heart Transplantation

Despite improvement in thyroid function, his plastic bronchitis and heart function continued to deteriorate, leading to Fontan failure. Given his progressive clinical decline, he was listed for transplantation and successfully completed a heart transplant at age 15.

## 3. Discussion

This case represents a rare and instructive example of sequential iodine-induced thyroid dysfunction in a pediatric patient. Initially, the patient developed hypothyroidism due to failure to escape from the Wolff–Chaikoff effect, a physiologic response in which excess iodine acutely suppresses thyroid hormone synthesis. In healthy individuals, this inhibition is transient, with the thyroid “escaping” from suppression within days. However, in susceptible individuals, escape may fail, resulting in sustained hypothyroidism. Later, following re-exposure to iodinated contrast, our patient developed Jod-Basedow syndrome, or iodine-induced hyperthyroidism. To our knowledge, this is the first reported pediatric case demonstrating both iodine-induced hypothyroidism and hyperthyroidism in the same patient after repeated iodinated contrast exposure.

Iodine-induced hypothyroidism has been well described in both adult and pediatric patients. Cases have been reported in neonates exposed to iodinated contrast during intralymphatic imaging [1], as well as in infants with congenital heart disease exposed to iodine through contrast agents, topical antiseptics, or iodine-impregnated dressings postoperatively [2]. More broadly, hypothyroidism after contrast exposure has been documented in children and adults, often in the setting of underlying thyroid or renal dysfunction, or exposure to multiple procedures [3–5].

Conversely, Jod-Basedow syndrome is a less common but important cause of thyrotoxicosis, particularly in patients with underlying thyroid dysfunction. Jod-Basedow phenomenon is more likely in those with nodular goiter, subclinical hyperthyroidism, and in regions with chronic iodine deficiency, but our patient did not have any of these characteristics [6, 7]. Prior reports have described Jod-Basedow syndrome following iodinated contrast exposure in both previously healthy individuals [8] and those with pre-existing thyroid dysfunction.

While Jod-Basedow syndrome has been recognized in adult patients undergoing imaging studies or procedures involving iodinated contrast exposure [9, 10], it has been less frequently reported in pediatric populations, particularly those with congenital heart disease. This case further supports existing literature that thyroid dysfunction may manifest even in young patients following iodine exposure [8], necessitating careful monitoring. Rapid onset of thyrotoxicosis after iodine exposure has been well documented, with some cases occurring within hours [11]. Our patient developed symptoms within 48 h, aligning with prior cases of iatrogenic hyperthyroidism.

Beyond iodinated contrast, various iodine-containing substances have been linked to Jod-Basedow syndrome, including oral iodine solutions [12, 13], peritoneal dialysis with povidone-iodine [14], as well as topical povidone-iodine dressings[15]. This reinforces the need for heightened awareness of potential iodine-related thyroid dysfunction, particularly in high-risk patients.

Children with complex congenital heart disease often undergo repeated imaging and interventional procedures, increasing their cumulative iodine exposure. This case emphasizes the importance of thyroid function screening prior to, and monitoring after, iodinated contrast use in vulnerable populations. Given the potential for rapid onset and severe cardiovascular effects in patients with Fontan physiology, early recognition and management of thyroid dysfunction are crucial to optimizing outcomes.

## 4. Conclusion

This case illustrates the rare occurrence of iodine-induced hypothyroidism, followed by hyperthyroidism in an adolescent with congenital heart disease. The initial development of hypothyroidism likely resulted from failure to escape the Wolff–Chaikoff effect, followed months later by Jod-Basedow syndrome after re-exposure to iodinated contrast.

While each condition is recognized individually, this is the first reported pediatric case to exhibit both iodine-induced hypothyroidism and hyperthyroidism occurring in succession, in a patient with complex cardiac disease. In addition, previous cases of Jod-Basedow syndrome have been reported with cardiac effects in the adult population [16], as well as in previously healthy pediatric patients [8], but this is the first documented case of Jod-Basedow syndrome in a pediatric patient with complex congenital heart disease.

Given the increasing reliance on iodinated contrast imaging in patients with congenital heart disease, this case underscores the need for careful thyroid function monitoring before and after iodine exposure. Clinicians should maintain a high index of suspicion for iodine-induced hypo- and hyperthyroidism, particularly in high-risk populations. In this patient, early recognition and treatment of hyperthyroidism with methimazole led to resolution of hyperthyroidism; however, his overall clinical course continued to decline, ultimately requiring heart transplantation. This case highlights the intricate relationship between endocrine and cardiac health in medically complex patients and reinforces the importance of individualized surveillance strategies to mitigate endocrine-related complications in this vulnerable population.

## Data Availability

The clinical data supporting this case report were obtained from the patient's electronic medical record and are not publicly available due to privacy and ethical restrictions. Further details may be available from the corresponding author upon reasonable request and with appropriate institutional approvals.
